# Hippo component YAP promotes focal adhesion and tumour aggressiveness via transcriptionally activating THBS1/FAK signalling in breast cancer

**DOI:** 10.1186/s13046-018-0850-z

**Published:** 2018-07-28

**Authors:** Jie Shen, Beibei Cao, Yatao Wang, Chenshen Ma, Zhuo Zeng, Liang Liu, Xiaolan Li, Deding Tao, Jianping Gong, Daxing Xie

**Affiliations:** 1 Molecular Medicine Center, Tongji Hospital, Tongji Medical College, Huazhong University of Science and Technology, 1095 Jiefang Av., Wuhan, Hubei 430030 People’s Republic of China; 2 Department of Surgery, Tongji Hospital, Tongji Medical College, Huazhong University of Science and Technology, 1095 Jiefang Av., Wuhan, Hubei 430030 People’s Republic of China

**Keywords:** Breast cancer, Focal adhesion, YAP, THBS1, FAK

## Abstract

**Background:**

Focal adhesion plays an essential role in tumour invasiveness and metastasis. Hippo component YAP has been widely reported to be involved in many aspects of tumour biology. However, its role in focal adhesion regulation in breast cancer remains unexplored.

**Methods:**

Tissue microarray was used to evaluate YAP expression in clinical breast cancer specimens by immunohistochemical staining. Cell migration and invasion abilities were measured by Transwell assay. A cell adhesion assay was used to measure the ability of cell adhesion to gelatin. The focal adhesion was visualized through immunofluorescence. Phosphorylated FAK and other proteins were detected by Western blot analysis. Gene expression profiling was used to screen differently expressed genes, and gene ontology enrichment was performed using DAVID software. The gene mRNA levels were measured by quantitative real-time PCR. The activity of the THBS1-promoter was evaluated by dual luciferase assay. Chromatin immunoprecipitation (ChIP) was used to verify whether YAP could bind to the THBS1-promoter region. The prediction of potential protein-interaction was performed with the String program. The ChIP sequence data of TEAD was obtained from the ENCODE database and analysed via the ChIP-seek tool. The gene expression dataset (GSE30480) of purified tumour cells from primary breast tumour tissues and metastatic lymph nodes was used in the gene set enrichment analysis. Prognostic analysis of the TCGA dataset was performed by the SurvExpress program. Gene expression correlation of the TCGA dataset was analysed via R2: Genomics Analysis and Visualization Platform.

**Results:**

Our study provides evidence that YAP acts as a promoter of focal adhesion and tumour invasiveness via regulating FAK phosphorylation in breast cancer. Further experiments reveal that YAP could induce FAK phosphorylation through a TEAD-dependent manner. Using gene expression profiling and bioinformatics analysis, we identify the FAK upstream gene, thrombospondin 1, as a direct transcriptional target of YAP-TEAD. Silencing THBS1 could reverse the YAP-induced FAK activation and focal adhesion.

**Conclusion:**

Our results unveil a new signal axis, YAP/THBS1/FAK, in the modulation of cell adhesion and invasiveness, and provides new insights into the crosstalk between Hippo signalling and focal adhesion.

**Electronic supplementary material:**

The online version of this article (10.1186/s13046-018-0850-z) contains supplementary material, which is available to authorized users.

## Background

Although great achievements have been made in the areas of screening, diagnosis and therapy, breast cancer is still the leading cause of cancer-related deaths in women worldwide [1]. In breast cancer patients, metastasis at distant sites, rather than primary tumour, is the major obstacle of treatment and the main cause of cancer lethality [2]. Metastasis is a long, sequential process, in which the interaction between cancer cells and the tumour extracellular matrix (ECM) is essential [3]. Cell-ECM crosstalk plays a key role in regulating tumour cell motility and invasiveness through numerous cellular biomechanics, such as focal adhesion, membrane remodelling, actin protrusion, actomyosin contraction, and cell motility signalling pathways [4]. Among these, focal adhesion has been revealed to be a crucial determinant of cell migration and plays an important role in promoting tumour cell invasion [5].

Focal adhesion (FA) is a subcellular structure which provides strong adhesion to the ECM and acts as a scaffold for many signalling pathways involving integrin or the mechanical force exerted on cells [6]. Recent studies have revealed the dynamic cycle of “FA assembly–cytoskeleton remodelling–FA disassembly”, which allows cells to achieve motility, and the dysregulation of FA is considered to be an essential step in tumour invasion [5, 7]. Many components of FA are tyrosine kinases and their substrates, of which focal adhesion kinase (FAK, also known as PTK2) has been demonstrated to be a major participant in FA dynamics [8]. After integrin engagement, FAK is recruited and phosphorylated at Tyr397 [9]; the phosphorylated FAK leads to the recruitment of other signalling molecules and promotes the assembly of FA complexes [8]. In addition, there is also evidence showing that FAK is necessary in FA disassembly [10]. As a key regulator of FA, FAK plays an oncogenic role in a wide range of human cancers [11]. Increased FAK expression and activity are often correlated with metastasis and poor prognosis [12–14]. Previous research has proven the correlation between FAK activation and metastasis in breast cancer [15]. Disrupting FAK could slow metastasis formation of mammary tumours [16, 17]; thus, it has been selected as a potential therapeutic target for aggressive breast cancers (reviewed in [18]). Although the significance of focal adhesion and FAK in breast malignancy metastasis has been widely reported, it is still unclear how FA is regulated in tumour progression.

Over the past decade, Hippo signalling has been proven to be a master regulator network in many aspects of tumour biology [19, 20]. Yes-associated protein (YAP) acts as the main effector of the Hippo pathway and triggers downstream biological effects through inducing target gene transcription via interacting with related transcription factors, especially TEA domain family members (TEADs) [21]. YAP has been considered to be an oncogene in breast cancer, and its dysregulation often leads to tumour aggressiveness and metastasis [22, 23]. Recent studies have uncovered the critical role of YAP in the regulation of actin dynamics and cell motility [24, 25]. This evidence indicates a potential relationship between Hippo signalling and tumour metastasis; however, the concrete mechanism still remains to be explored.

This current study focuses on the role of YAP in FA regulation and tumour metastasis in breast cancer. In this research, we have revealed the potential relationship between YAP activation and tumour metastasis in clinical breast tumour specimens. Through in vitro experiments we have observed that YAP could significantly promote FA formation and FAK activation in breast cancer cell lines. Furthermore, we have validated that the YAP-TEAD interaction is essential for these biological effects. Using gene expression profiling and the ENCODE database, we have identified Thrombospondin 1 (THBS1), a previously reported FAK stimulator [26–28], as a direct transcriptional target of Hippo signalling. We have further demonstrated that YAP/TEAD could increase THBS1 expression to promote FAK phosphorylation and FA formation, leading to the activation of tumour cell migration and invasiveness. Collectively, our findings revealed a novel function of Hippo signalling in inducing FAK activation and focal adhesion formation to promote breast cancer aggressiveness and metastasis.

## Materials and methods

### Tissue microarray and immunohistochemistry (IHC)

A human breast cancer tissue microarray of 104 cases of paired primary lesion/lymphatic metastasis (US Biomax, Cat. #BR20837a) was used to evaluate the expression of YAP in primary and metastatic tissue. The slide was dewaxed, rehydrated and heated in sodium citrate buffer (0.01 M, pH 6.0) for antigen retrieval. Endogenous peroxidase was then inhibited with 3% hydrogen peroxide with 0.1% sodium azide for 30 min and non-specific staining was blocked by incubation in 5% bovine serum albumin for 2 h. The slide was then incubated in 1:100 diluted YAP antibody (Cell Signaling Technology, Cat. #4912) at 4 °C overnight and subsequently with biotinylated secondary antibody for 2 h. The DAB Horseradish Peroxidase Color Development Kit (Wuhan BosterBio Co. Ltd., Cat. #AR1022) was used for immunostaining, and counterstain was performed by haematoxylin staining. The results were analysed under a microscope.

The expression level of YAP was evaluated by the IHC score, which was calculated by multiplying a proportion score and an intensity score, and was categorized as level 1 (IHC score 0–3), level 2 (IHC score 4–6) or level 3 (IHC score greater than 6). The proportion score reflected the fraction of positive-stained cells (0, none; 1, ≤10%; 2, 10% to ≥25%; 3, > 25 to 50%; 4, > 50%), and the intensity score revealed the staining intensity (0, no staining; 1, weak; 2, intermediate; 3, strong). The nucleus localization of YAP was measured by a nucleus score. The nucleus score represented the fraction of positive-stained nuclei (0 = 0–10%; 1 = 11–30%; 2 = 31–70%; 3 = 71–100%). The cytoplasm expression of YAP was evaluated by a cytoplasmic score. The cytoplasmic score was calculated by multiplying the intensity of cytoplasmic staining (no staining = 0, weak staining = 1, moderate staining = 2, strong staining = 3) and the extent of stained cells (0 = 0–10%; 1 = 11–30%; 2 = 31–70%; 3 = 71–100%).

### Cell culture and transfection

Human breast cancer cell lines MDA-MB-231, MCF7 and human embryonic kidney cell line HEK293T were purchased from American Type Culture Collection (ATCC). The HEK293T and MCF7 cell lines were routinely cultured in Dulbecco’s modified Eagle medium (DMEM, KeyGEN), and the MDA-MB-231 cell line was maintained in Leibovitz’s L-15 medium (L15, KeyGEN). DMEM and L15 culture media were supplemented with 10% foetal bovine serum (MULTICELL, Cat. #086–150) and 1% penicillin/streptomycin (KeyGEN). MCF7 and HEK293T cells were cultured at 37 °C in a 5% CO_2_ incubator, while MDA-MB-231 cells were cultured at 37 °C in a 100% air incubator, according to the ATCC instructions.

Small-interfering RNAs (siRNAs) targeting YAP and THBS1 were designed and synthesized by Guangzhou RiboBio Co. Ltd. The sequences of the siRNAs are described in Additional file 1: Table S1. The siRNA transfections were performed using Lipofectamine® 2000 transfection reagent (Thermo Fisher, Cat. #11668019), according to the manufacturer’s protocol. Non-targeting siRNAs (siNCs) were used for the negative control. After 48 h, cell biological and biochemical experiments were performed.

The plasmids pcDNA3.1-YAP, pcDNA3.1-YAP-S127A (FLAG-tagged) and pcDNA3.1-YAP-S94A (GFP-tagged) were previously constructed and used for the overexpression of YAP and its mutants. After transfection, the cells were treated with 500 μg/ml G418 (Santa Cruz Biotechnology, Cat. #sc-29,065) for 4 weeks to obtain stable cell lines. Empty vector was used as a negative control.

### Transwell migration and invasion assay

Transwell plates (24-well, pore size 8 μm (Corning, Cat. #3422)) were used for the transwell assay. For the migration assay, 1*10^5^ cells were harvested in 100 μl of serum-free culture medium and added into the upper chamber, without Matrigel. For the invasion assay, transwell filters were pre-coated with 30 μl of 1:8 diluted Matrigel (BD, Cat. #356234) prior to the addition of the cell suspension. Next, 600 μl of 30% foetal bovine serum medium was placed into the bottom compartment of the chamber as a source of chemo-attractant. After 24 h of culturing, the cells that crossed the inserts were fixed and strained with crystal-violet. Migrated cells were photographed and counted via an inverted microscope (100X magnification).

### Cell adhesion assay

The cell adhesion assay was performed as previously described [29]. Briefly, 2*10^5^ cells per well were seeded into 24-well plates on coverslips that were pre-coated with 1% sterile gelatin (Sigma, Cat. #G-2500) and exposed to different treatments. After 2 h of culturing, the plates were gently washed with PBS to remove the non-adherent cells. The attached cells were then fixed with 4% formaldehyde and stained with Wright’s-Giemsa. Attached cells were photographed and counted using a microscope with 100X magnification.

### Immunofluorescence

Twenty thousand cells per well were seeded into 24-well plates on 12 mm coverslips that were pre-coated with 1% sterile gelatin (Sigma, Cat. #G-2500) and exposed to different treatments. After 24 h of culturing, the cells were fixed with 4% paraformaldehyde and permeabilized with 0.1% Triton X-100/PBS. Blocking of nonspecific staining was achieved by incubation in 5% bovine serum albumin/PBS for 2 h. Subsequently, the cells were incubated overnight at 4 °C with anti-paxillin antibody (Abcam, Cat. #ab32084) at a dilution of 1:100, followed by incubation in a solution of fluorescently labelled secondary antibody (1:100) (Abbkine, Cat. #A24221, A23620) and 1:100 phalloidin (Life Technologies, Cat. #A22287) for 2 h. Nuclei was strained by DAPI, and coverslips were placed face down onto a drop of anti-fading mounting medium on a microscope slide. Images were captured via a confocal laser scanning microscope with 400X magnification. Each experiment was performed in triplicate.

### Western blot assay

Total protein was extracted with NP40 lysis buffer with the addition of phenylmethylsulfonyl fluoride and protein phosphatase inhibitor cocktail (Cell Signaling Technology, Cat. #5870) and was separated on 10% SDS-PAGE gels. After electrophoresis, the separated protein bands were transferred onto polyvinylidene fluoride membranes (Millipore, Cat. #IPVH00010) and blocked in 5% non-fat milk for 1 h at room temperature. The membranes were then incubated with the primary antibodies against YAP (Cell Signaling Technology, Cat. #4912), FAK (Abclonal, Cat. #A11131), pY397-FAK (Abclonal, Cat. #AP0302), THBS1 (Abclonal, Cat. #A2125) and GAPDH (Santa Cruz Biotechnology, Cat. #sc-32,233) at a diluted ratio of 1:1000 overnight at 4 °C. After washing three times, the membranes were incubated in 1:5000 horseradish peroxidase-linked secondary antibodies at room temperature for 2 h. Finally, the membranes were washed three times and were visualized using an ECL Kit (Thermo Fisher, Cat. #34096).

### Gene expression profiling

Total RNA of MCF7-vector and MCF7-YAP1-S127A were freshly extracted using TRIzol reagent (Takara, Cat. #9108). RNA quantity and integrity were assessed using a NanoDrop ND-2000 (Thermo Scientific) and an Agilent Bioanalyzer 2100 (Agilent Technologies). The gene expression profiling was conducted by Shanghai Oebiotech Corporation using the Agilent SurePrint G3 Human Gene Expression v3 Panel (Agilent, CA, USA). All data were analysed according to the manufacturer’s protocol. Differentially expressed genes were then identified by fold change. The threshold set for up- and downregulated genes was a fold change greater than 2.

### Quantitative real-time PCR (qRT-PCR)

Total RNA was extracted using TRIzol (Takara, Cat. #9108), according to the manufacturer’s protocol. Reverse-transcription was performed to obtain cDNA using a PrimeScript™ RT Master Mix Reagent Kit (Takara, Cat. #RR036A), and quantitative real-time PCR was carried out using a TB Green™ Premix Ex Taq™ II Kit (Takara, Cat. #RR820A) according to the manufacturer’s protocol. GAPDH gene expression was used as an endogenous control, and the results from qRT-PCR were analysed though the comparative Ct method (2^-ΔΔCt^). The primer sequences used in this research are provided in Additional file 2: Table S2. Each experiment was performed in triplicate.

### Dual luciferase assay

A total of 100 ng of pGL3-Basic plasmid (Promega, Cat. #E1751) with inserts of the THBS1 promoter sequence (TSS: − 2000 ~ + 50) were co-transfected into HEK293T and MCF7 cells using Lipofectamine® 2000 transfection reagent (Thermo Fisher, Cat. #11668019) along with 200 ng of YAP/YAP-mutant construct and 10 ng of Renilla luciferase pRL-TK plasmid (Promega, Cat. #E2241). After 48 h, the dual luciferase assay was performed using the Dual-Luciferase® Reporter Assay System (Promega, Cat. #E1910), according to the manufacturer’s protocol. Luciferase activity was measured as the ratio of firefly luciferase signal to Renilla luciferase signal. All measurements were normalized to the control group alone. Each experiment was performed in triplicate.

### Chromatin immunoprecipitation (ChIP)

After transfecting with empty vector or YAP1-S127A plasmid for 48 h, the MCF7 cells were harvested, and ChIP experiments were performed using the SimpleChIP Enzymatic Chromatin IP Kit (Cell Signaling Technology, Cat. #9003), according to the manufacturer’s protocol. A total of 500 μl of diluted cross-linked chromatin was incubated overnight with 5 μg of mouse monoclonal anti-YAP antibody (Cell Signaling Technology, Cat. #14074) or with 1 μg of normal mouse IgG (Cell Signaling Technology, Cat. #2729) at 4 °C. The THBS1 promoter sequence (primers: F: ACCGACTTTTCTGAGAAG, R: GCAACTTTCCAGCTAGAA) were quantified by PCR and analysed by 2% agarose gel electrophoresis with a 100 bp DNA marker.

### Public database and bioinformatics analysis

The gene expression dataset (GSE30480, [30]) of purified tumour cells from 14 primary breast tumour tissues and 6 metastatic lymph nodes was obtained from the Gene Expression Omnibus database and was used in gene set enrichment analysis (GSEA) (http://software.broadinstitute.org/gsea/) [31]. The prognostic analysis of TCGA breast invasive carcinoma dataset was performed by the SurvExpress program (http://bioinformatica.mty.itesm.mx:8080/Biomatec/SurvivaX.jsp) [32]. The ChIP-sequence data of TEAD4 in the MCF7 cell line was downloaded from the ENCODE project (http://genome.ucsc.edu/ENCODE/downloads.html) (GSM1010860) and was analysed via the ChIPseek online tool (http://chipseek.cgu.edu.tw/) [33]. The String database (http://www.string-db.org/) [34] was used for protein interaction analysis. The gene expression correlations were revealed by the R2: Genomics Analysis and Visualization Platform (http://r2.amc.nl) using the TCGA invasive carcinoma dataset. Gene ontology enrichment was performed by the DAVID software (https://david.ncifcrf.gov/tools.jsp).

### Statistical analysis

Statistical analysis was performed with the SPSS software package (version 19.0 for Windows; IBM, USA). All continuous data are presented as the mean ± SD and statistically analysed with Student’s t-test (two-tailed) and analysis of variance (ANOVA). A *p*-value less than 0.05 was considered to be statistically significant.

## Results

### YAP overexpression and activation were associated with lymphatic metastasis and poor prognosis in breast cancer patients

**Fig. 1:**
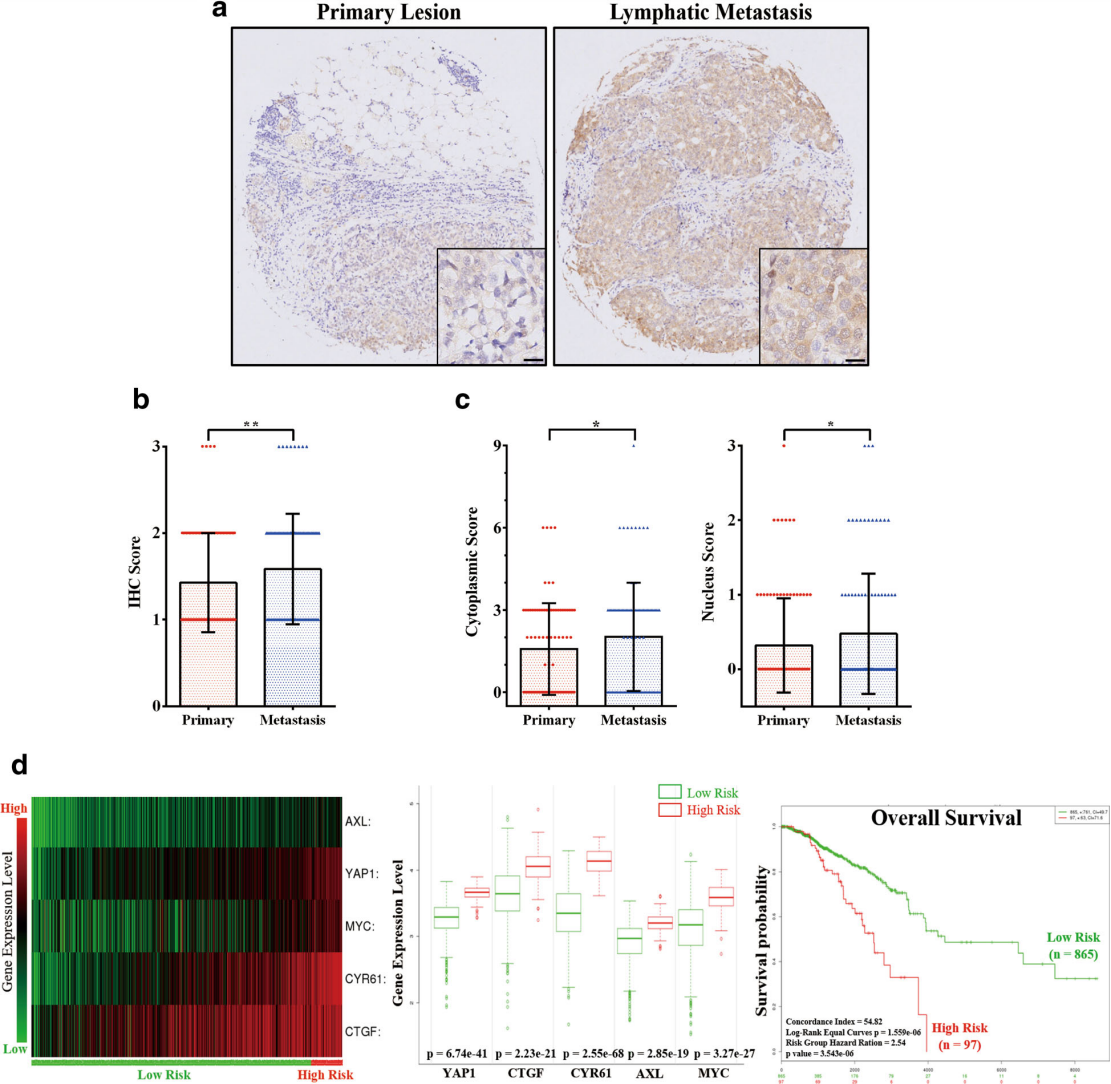
YAP overexpression and activation were associated with lymphatic metastasis and poor prognosis in breast cancer patients. (**a**) Immunohistochemistry staining of YAP protein in paired primary and lymphatic metastatic specimens from one breast cancer patient. Lymphatic metastasis revealed a higher expression level of YAP. Scale bar: 20 μm. (**b**) Immunohistochemistry staining score (IHC score) of YAP expression in 101 paired primary/lymphatic metastatic breast cancer specimens from a breast cancer tissue microarray. The expression level of YAP was significantly higher in lymphatic metastases than in primary lesions (**p < 0.01 by paired Student’s t-test). Primary: primary lesion; Metastasis: lymphatic metastasis. (**c**) Immunohistochemistry cytoplasm expression (cytoplasmic score, left panel) and nucleus accommodation (nucleus score, right panel) of YAP in the 101 paired primary/lymphatic metastatic breast cancer specimens. The cytoplasm expression and nucleus accumulation of YAP was significantly higher in lymphatic metastases than in primary lesions (*p < 0.05 by paired Student’s t-test). Primary: primary lesion; Metastasis: lymphatic metastasis. (**d**) Analysis of TCGA breast invasive carcinoma dataset (*n* = 962) via SurvExpress program. Left: Heat map summarizing the expression values of YAP and its target genes (CTGF, CYR61, AXL and MYC) in breast cancer specimens from the TCGA dataset. Patients were sorted by prognostic index and divided into “Low Risk” and “High Risk” groups, according to the “Maximized Risk Groups” algorithm (see reference [32]). Middle: patients in the “High Risk” group presented a significantly higher expression level of YAP and its downstream genes (*p* < 0.01). Right: Kaplan-Meier analysis revealed that patients in the “High Risk” group suffered from poor prognosis (*p* < 0.01)

To validate the relationship between YAP expression and metastasis in breast cancer patients, a paraffin-embedded tissue array containing 104 paired primary/lymphatic metastatic clinical breast cancer specimens was obtained. Due to dropping, moving and wrinkling during the experiments 3 cases were discarded, and the remaining 101 paired specimens were analysed. Through immunohistochemistry staining, we found that the YAP expression level was relatively higher in lymphatic metastases than in primary lesions (Fig. 1a, IHC score shown in Fig. 1b). In addition, the IHC cytoplasmic and nucleus scoring showed that the YAP protein had a higher level of cytoplasm expression and nucleus accumulation in lymphatic metastases (Fig. 1c). Furthermore, gene set enrichment analysis was performed on a gene expression profile of purified tumour cells from 14 primary breast tumours and 6 metastatic lymph nodes that was available from the GSE database (GSE30480, [30]). The results revealed that the YAP conserved signature was enriched in metastatic lymph nodes, with statistical significance (Additional file 3: Figure S1). This evidence indicated that YAP expression and activation was positively associated with lymphatic metastasis in breast cancer.

For the purpose of determining whether YAP overexpression and activation were associated with a poor prognosis in breast cancer, we used SurvExpress [32] to evaluate the expression level of YAP and its downstream genes (CTGF, CYR61, AXL and MYC [35, 36]) in the TCGA invasive carcinoma dataset (Fig. 1d, left, patients were sorted in the ascending order of prognostic index). Through the SurvExpress program, patients in the TCGA dataset were divided into “Low Risk” and “High Risk” groups according to the prognostic index. Patients in the “High Risk” group presented a significantly higher expression level of YAP and its downstream genes and suffered from poor prognosis (Fig. 1d, middle and right). Therefore, overexpression and activation of YAP was supposed to be a biomarker of poor survival in breast cancer patients.

### YAP induced cell migration, invasion and focal adhesion in breast cancer cell lines

**Fig. 2:**
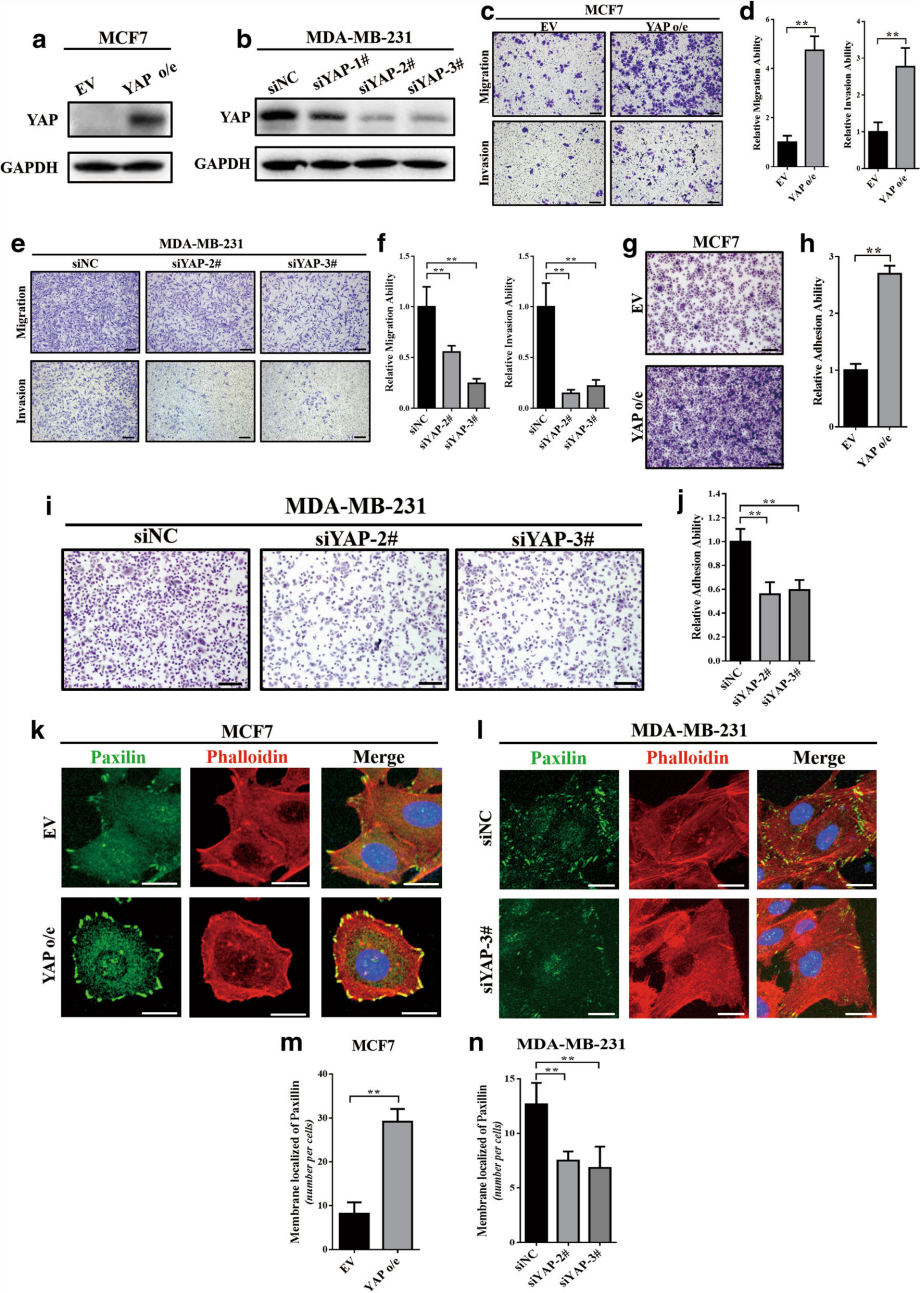
YAP was able to induced cell migration, invasion and focal adhesion in breast cancer cell lines**.** (**a**) Western blot verified the overexpression of YAP in MCF7 cells. EV: empty vector; o/e: overexpression. (**b**) Western blot verified the knockdown of YAP in MDA-MB-231 cells via a collection of siRNAs; siYAP-#2 and siYAP-#3 has relatively high knockdown efficiency, thus these two siRNAs were used in this research. (**c**, **d**) Transwell assay showing that overexpression of YAP induced cell migration and invasion ability in MCF7 cells. The experiment was performed in triplicate. **p < 0.01 by Student’s t-test. Scale bar: 100 μm. (**e**, **f**) Transwell assay showing that knockdown of YAP significantly inhibited cell migration and invasion ability in MDA-MB-231 cells. The experiment was performed in triplicate. ** *p* < 0.01 by ANOVA test. Scale bar: 100 μm. (**g**, **h**) Overexpression of YAP induced MCF7 cell adhesion to gelatin. The attached cells were stained with Wright’s-Giemsa and are shown in (**g**). The experiment was performed in triplicate. ** *p* < 0.01 by Student’s t-test. Scale bar: 100 μm. (**i**, **j**) Knockdown of YAP significantly inhibited MDA-MB-231 cell adhesion to gelatin. The attached cells were stained with Wright’s-Giemsa and are shown in (**i**). The experiment was performed in triplicate. ** *p* < 0.01 by Student’s t-test. Scale bar: 100 μm. (**k**) Overexpression of YAP induced focal adhesions in MCF7 cells. Focal adhesions were visualized by co-localization of paxilin (green) and F-actin (stained with phalloidin, red). Nuclei were counterstained with DAPI (blue). Scale bar: 20 μm. (**l**) Knockdown of YAP expression inhibited focal adhesions in MDA-MB-231 cells. Scale bar: 20 μm. (**m**) Quantification of the membrane-localized paxilin in (**k**). The experiment was performed in triplicate. ** *p* < 0.01 by Student’s t-test. (**n**) Quantification of the membrane-localized paxilin in (**l**). The experiment was performed in triplicate. ** *p* < 0.01 by ANOVA test

Previously, we examined the expression levels of YAP protein in 4 breast cancer cell lines (MCF7, T47D, MDA-MB-231 and MDA-MD-468) and revealed that YAP was overexpressed in MDA-MB-231, MDA-MB-468 and T47D cell lines, while it was relatively low expressed in the MCF7 cell line (data not shown). MDA-MB-231 and MCF7 have been reported to have a high and low metastatic potential, respectively [37]; therefore, these two cell lines were selected for further study. MCF7 was stably transfected with pcDNA3.1-YAP plasmid to overexpress YAP protein (Fig. 2a), and a collection of siRNAs was used to knockdown endogenous YAP expression in MDA-MB-231 cells (Fig. 2b). As shown in Fig. 2b, siYAP-#2 and siYAP-#3 demonstrated a relatively high knockdown efficiency, thus these two siRNAs were used in this research. The transwell assay revealed that the expression level of YAP was positively correlated with cell migration and invasion ability, both in MCF7 (Fig. 2c, d) and MDA-MB-231 (Fig. 2e, f) cells.

Due to the important role of cell adhesion in cancer invasion and metastasis [3], we next validated whether YAP could regulate the cell adhesion ability. The overexpression of YAP could significantly induce MCF7 cell adhesion to gelatin (Fig. 2g, h), while knockdown of YAP in MDA-MB-231 cells significantly inhibited cell adhesion ability (Fig. 2i, j). Interestingly, through immunofluorescence we observed that the number of focal adhesions, an important sub-cellular structure that mediates the regulatory effects of a cell to ECM adhesion [38], was strongly associated with overexpression of YAP in MCF7 (Fig. 2k, m) and was significantly decreased by YAP knockdown in MDA-MB-231 cells (Fig. 2l, n). These results indicated that YAP could increase cell migration, invasion and adhesion abilities and induce focal adhesion formation in breast cancer cell lines.

### YAP-TEAD interaction was essential for tumour cell invasiveness and focal adhesion formation

**Fig. 3:**
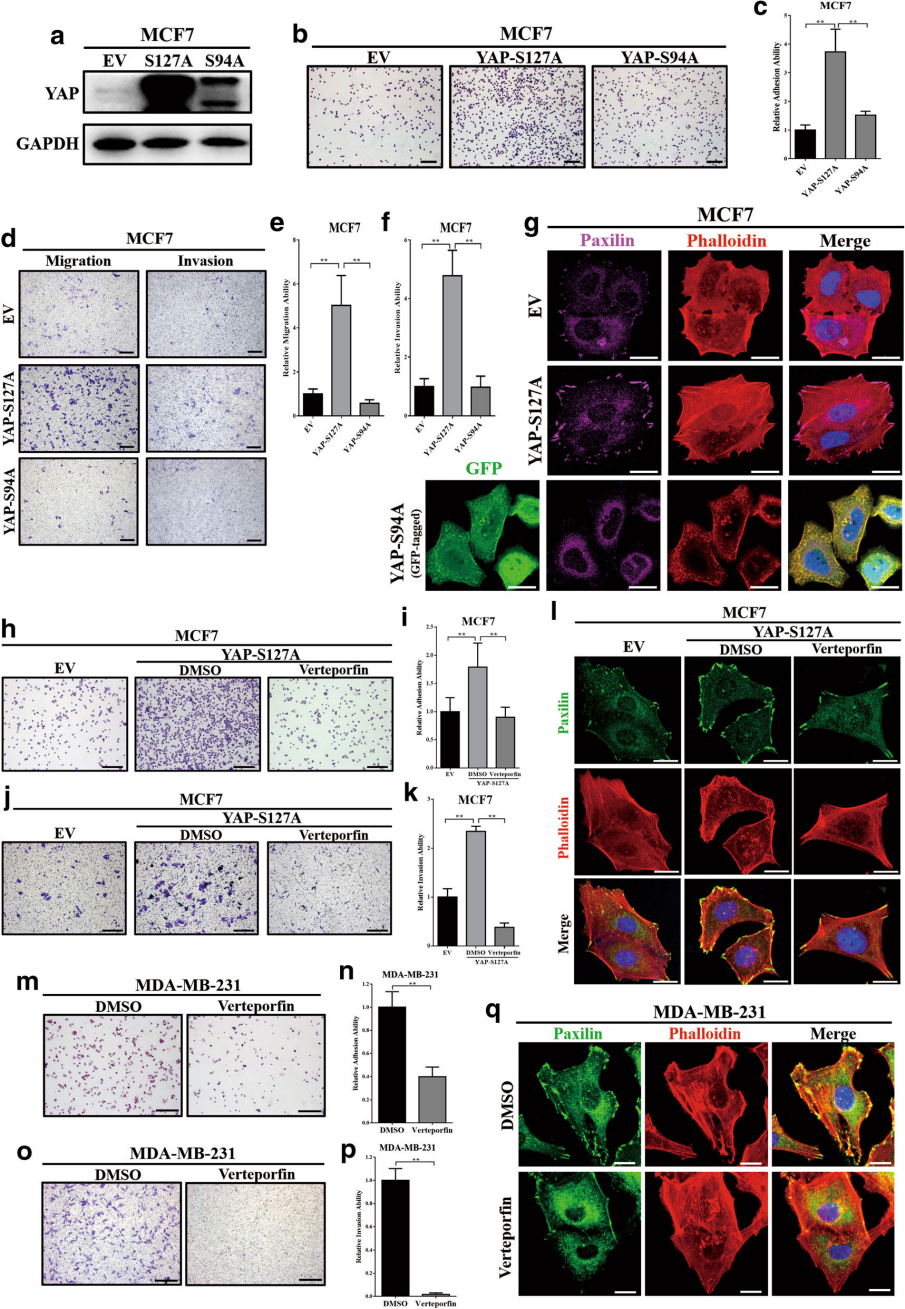
YAP-TEAD interaction was essential for tumour cell invasiveness and focal adhesion formation. (**a**) Western blot verified the overexpression of two YAP mutants, YAP-S127A (FLAG-tagged) and YAP-S94A (GFP-tagged) in MCF7 cells. EV: empty vector; S127A: YAP constitutively activated mutant (YAP1-S127A); S94A: YAP TEAD-binding domain mutant (YAP-S94A). (b) (c) Cell adhesion assays showed that ectopic expression of YAP-S127A, rather than YAP-S94A, induced MCF7 cell adhesion to gelatin. The experiment was performed in triplicate. ** *p* < 0.01 by ANOVA test. Scale bar: 100 μm. (**d, e**, **f**) Transwell assays showed that compared with the YAP-S94A mutant, YAP-S127A could significantly induce cell migration and invasion ability in MCF7 cells. The experiment was performed in triplicate. ** *p* < 0.01 by ANOVA test. Scale bar: 100 μm. (**g**) Ectopic expression of YAP-S127A, rather than YAP-S94A, induced focal adhesions in MCF7 cells. Focal adhesions were visualized by co-localization of paxilin (stained with Dylight 649, violet) and F-actin (stained with phalloidin, red). Nuclei were counterstained with DAPI (blue). GFP is represented as green. Scale bar: 20 μm. (**h**, **i**) Representative images of MCF7-YAP-S127A cell adhesion to gelatin after treatment with verteporfin at a dose of 10 μM for 24 h (DMSO was used as negative control). Verteporfin significantly inhibited cell adhesion ability of MCF7 cells expressing YAP-S127A mutant. The experiment was performed in triplicate. **p < 0.01 by ANOVA test. Scale bar: 100 μm. (**j**, **k**) Transwell assays showed that verteporfin significantly inhibited invasion ability of MCF7-YAP-S127A cells. MCF7-YAP-S127A cells were treated with verteporfin at a dose of 10 μM for 24 h (DMSO was used as negative control) before transwell assays were performed. The experiment was performed in triplicate. **p < 0.01 by ANOVA test. Scale bar: 100 μm. (**l**) Verteporfin inhibited focal adhesions in MCF7-YAP-S127A cells. Cells were exposed to verteporfin (10 μM) or DMSO (negative control) for 24 h and then stained with paxilin (green). F-actin was stained with phalloidin (red). Nuclei were counterstained with DAPI (blue). Scale bar: 20 μm. (**m**, **n**) Verteporfin significantly inhibited cell adhesion ability in MDA-MB-231 cells. Cells were treated with verteporfin at a dose of 10 μM for 24 h before cell adhesion assays were performed. DMSO was used as negative control. The experiment was performed in triplicate. **p < 0.01 by Student’s t-test. Scale bar: 100 μm. (**o**, **p**) Verteporfin significantly inhibited invasion abilities in MDA-MB-231 cells. Cells were treated with verteporfin at a dose of 10 μM for 24 h (DMSO was used as negative control) before transwell assays were performed. The experiment was performed in triplicate. **p < 0.01 by Student’s t-test. Scale bar: 100 μm. (**q**) Treatment with verteporfin (10 μM) for 24 h decreased focal adhesions in MDA-MB-231 cells. Paxilin (green), F-actin (stained with phalloidin, red). Nuclei (blue). Scale bar: 20 μm

To investigate the regulation mechanism of YAP in cell invasiveness and focal adhesion formation, two pcDNA3.1-YAP mutant plasmids, FLAG-tagged-YAP-S127A (constitutive nuclei-accommodation mutant) and GFP-tagged-YAP-S94A (TEAD-binding domain mutant) were selected and stably transfected into MCF7 cells (Fig. 3a). Compared to the YAP-S94A mutant, ectopic expression of YAP-S127A in MCF7 could significantly increase the cell adhesion ability (Fig. 3b, c) and promoted cell migration and invasion (Fig. 3d-f). In addition, through immunofluorescence we found that expression of the YAP-S127A mutant, rather than YAP1-S94A, significantly increased focal adhesions in MCF7 (Fig. 3g). Thus, nucleus accommodation and TEAD-binding domain are required for the YAP-induced cell invasiveness and focal adhesion formation.

To further validate the essential role of the YAP-TEAD interaction in tumour invasiveness and focal adhesion, verteporfin (MCE, Cat. # HY-B0146), a small molecular inhibitor of the YAP-TEAD interaction [39], was employed. Verteporfin could significantly reverse YAP-S127A-induced cell adhesion (Fig. 3h, i) and invasion (Fig. 3j, k) in MCF7 cells. Furthermore, after treating with verteporfin, MCF7-YAP-S127A presented a reduction of focal adhesions (Fig. 3l). Additionally, verteporfin could also significantly inhibited cell adhesion (Fig. 3m, n), invasion (Fig. 3o, p) and focal adhesions (Fig. 3q) in MDA-MB-231 cells. Together these data suggest that the YAP-TEAD interaction was essential for tumour cell invasiveness and focal adhesion formation in breast cancer cell lines.

### YAP-TEAD promoted focal adhesion formation in breast cancer cell lines by inducing FAK phosphorylation

**Fig. 4:**
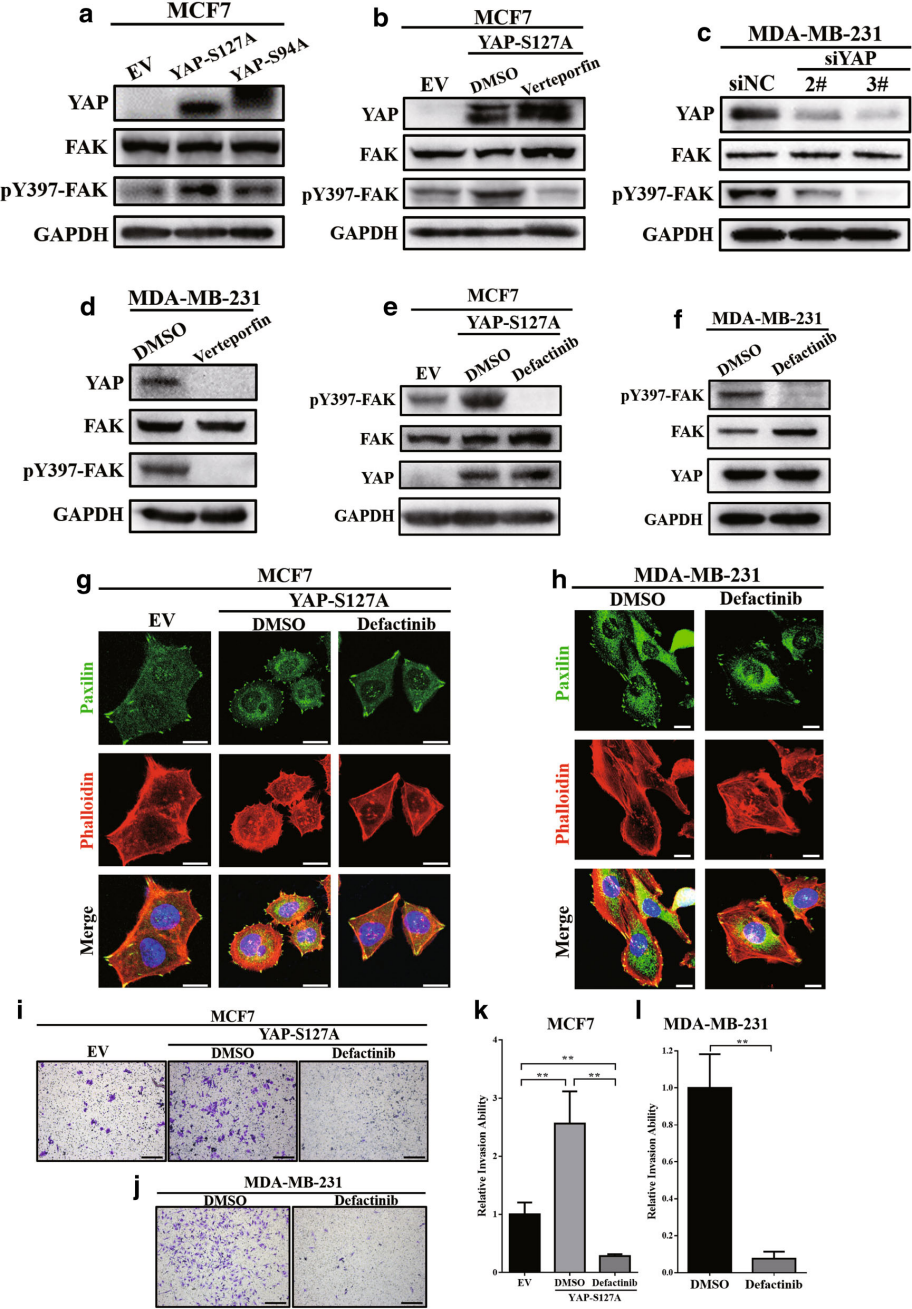
YAP-TEAD promoted focal adhesion formation in breast cancer cell lines through inducing FAK phosphorylation. (**a**) Western blot revealed that compared with the YAP-S94A mutant, overexpression of the YAP-S127A mutant could promote FAK Y397 phosphorylation. (**b**) Verteporfin reversed YAP-S127A-induced FAK phosphorylation in MCF7 cells. MCF7-YAP-S127A was treated with verteporfin at a dose of 10 μM for 24 h before the Western blot assay was performed. DMSO was used as a negative control. (**c**) Knockdown of endogenous YAP expression inhibited FAK Y397 phosphorylation in MDA-MB-231 cells. (**d**) Verteporfin inhibited FAK phosphorylation in MDA-MB-231 cells. Cells were treated with verteporfin at a dose of 10 μM for 24 h before Western blot assays were performed. DMSO was used as a negative control. (**e**, **f**) Western blot verified the inhibition of FAK Y397 phosphorylation via defactinib in MCF7-YAP-S127A (e) and MDA-MB-231 (**f**) cells. Cells were exposed to defactinib at a dose of 10 μM for 8 h before Western blot assays were performed. DMSO was used as a negative control. (**g**, **h**) Treatment with defactinib (10 μM) for 8 h decreased focal adhesions, both in MCF7-YAP-S127A (**g**) and MDA-MB-231 (**h**). DMSO was used as a negative control. Paxilin (green), F-actin (stained with phalloidin, red). Nuclei (blue). Scale bar: 20 μm. (**i**) Transwell invasion assays showed that exposure to defactinib (10 μM) could significantly reverse YAP-S127A-induced cell invasion in MCF7 cells. DMSO was used as a negative control. The experiment was performed in triplicate. Scale bar: 100 μm. (**j**) Exposure to defactinib (10 μM) significantly decreased cell invasion ability in MDA-MD-231 cells. DMSO was used as a negative control. The experiment was performed in triplicate. Scale bar: 100 μm. (**k**) Quantification of the relative invasion ability in (**i**). ** *p* < 0.01 by ANOVA test. (**l**) Quantification of the relative invasion ability in (**j**). ** *p* < 0.01 by Student’s t-test

Focal adhesion kinase (FAK, also known as PTK2) is a major component of focal adhesions, and the phosphorylation of FAK at Tyr397 has been demonstrated to be a main step in the assembly of focal adhesion complexes [8, 9]. In our study, we observed that the overexpression of YAP-S127A, rather than YAP-S94A, significantly induced FAK phosphorylation at Tyr397 in MCF7 (Fig. 4a). Applying verteporfin could significantly reverse FAK phosphorylation in MCF7 cells (Fig. 4b). Similarly, either knockdown of YAP expression or treatment with verteporfin significantly inhibited FAK Tyr397 phosphorylation in MDA-MB-231 cells (Fig. 4c, d). Interestingly, verteporfin also significantly decreased endogenous YAP protein levels in MDA-MB-231 (Fig. 4d). This may have been caused by the increasing proteasomal degradation of YAP due to the disruption of the YAP-TEAD complex; however, the specific mechanism requires further investigation.

Next, to determine the role of FAK phosphorylation in YAP-induced FA formation, a novel FAK inhibitor, defactinib (MedChemExpress, Cat. # HY-12289), was used to inhibit FAK Tyr397 phosphorylation in both MCF7-S127A (Fig. 4e) and MDA-MB-231 (Fig. 4f). As shown in Fig. 4g and Fig. 4h, after treating with defactinib, the number of focal adhesions was significantly decreased in MCF7-YAP-S127A and MDA-MB-231 cells. In addition, defactinib could also inhibit cell invasion in both MCF7-YAP-S127A (Fig. 4i, k) and MDA-MB-231 (Fig. 4j, l). This evidence revealed that FAK-Tyr397 phosphorylation was essential for YAP-TEAD regulated FA formation.

### YAP-TEAD transcriptionally promoted expression of FAK upstream regulatory factor, thrombospondin 1 (THBS1)

**Fig. 5:**
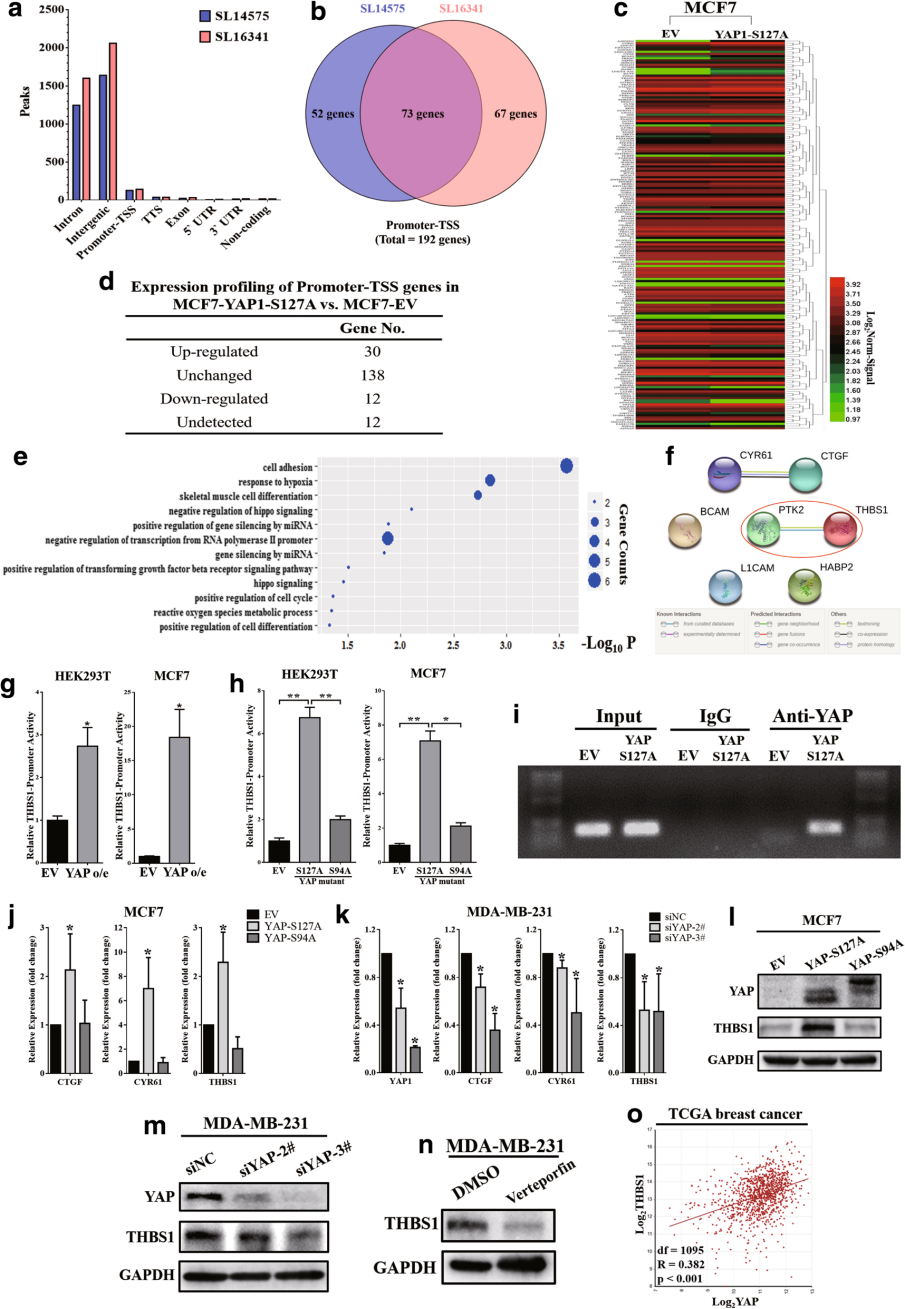
YAP-TEAD transcriptionally promoted expression of FAK upstream regulatory factor, thrombospondin 1 (THBS1). (**a**) Analysis of TEAD ChIP-sequence data of MCF7 cells from the ENCODE database (GSM1010860) via the ChIP-seek tool. SL14575 and SL16341 were two bio-replications of the ChIP-sequence data. (**b**) Peaks in promoter-TSS category from (**a**) were exacted and annotated. A total of 192 genes whose promoter was potentially combined with TEAD4 were identified. (**c**) Gene expression profiling was performed in MCF7 cells overexpressing the YAP-S127A mutant compared with empty vector. Expression values of the 192 genes from (**b**) were exacted and presented in a heat map. (**d**) The 192 genes were divided into four categories according to the expression fold change (FC) in MCF7-YAP-S127A vs. MCF7-EV cells. Upregulated: genes that were upregulated by the YAP-S127A mutant with a FC ≥ 2; Unchanged: genes with an expression fold change between the two groups of less than two; Downregulated: genes were downregulated by the YAP-S127A mutant with a FC ≥ 2; Undetected: genes that were not detected by the expression profiling. (**e**) Gene ontology analysis (biological processes) was performed for the 30 upregulated genes from (**d**). “Cell adhesion” was the first enrichment category and contained 6 genes. (**f**) Using the STRING program to analyse potential interactions between FAK (also known as PTK) and the 6 upregulated genes that were included in the “cell adhesion” category (THBS1, HABP2, L1CAM, BCAM, CYR61 and CTGF). THBS1 appeared to be highly correlated to FAK (confidence score: 0.849). (**g**) Dual luciferase reporter assays showed that THBS1 promoter activity could be significantly enhanced by YAP, both in HEK293T and MCF7 cells. *p < 0.05 by Student’s t-test. The experiments were performed in triplicate. (**h**) Dual luciferase reporter assays showed that compared to the YAP-S94A mutant, YAP-S127A could significantly increase THBS1 promoter activity in HEK293T and MCF7 cells. *p < 0.05 and **p < 0.01 by ANOVA. The experiments were performed in triplicate. (**i**) Through overexpressing the YAP-S127A mutant, the combination of YAP protein and THBS1 promoter was significantly increased in MCF7 cells. Chromatin and proteins were cross-linked, and mouse monoclonal anti-YAP antibodies were used for pulldown. The promoter of THBS1 was amplified and verified via agarose gel electrophoresis. Mouse IgG was used as a negative control. (**j**) Quantitative real-time PCR showed mRNA levels of YAP target genes (CTGF, CYR61) and THBS1 in MCF7-EV, MCF7-YAP-S127A and MCF7-S94A cells. The YAP-S127A mutant could significantly induce THBS1 and YAP target gene expression. GAPDH was used as an internal control. *p < 0.05 by ANOVA. The experiments were performed in triplicate. (**k**) Knockdown of endogenous YAP significantly downregulated THBS1, CTGF and CYR61 expression in MDA-MB-231 cells. GAPDH was used as an internal control. *p < 0.05 by ANOVA. The experiments were performed in triplicate. (**l**) Western blot showed that compared with the YAP-S94A mutant, overexpression of the YAP-S127A mutant significantly induced THBS1 expression. (**m**) Knockdown of endogenous YAP expression significantly decreased THBS1 protein levels in MDA-MB-231 cells. (**n**) Verteporfin could inhibit THBS1 expression in MDA-MB-231 cells. Cells were exposed to verteporfin (10 μM) for 24 h before the Western blot assay was performed. (**o**) THBS1 expression was positively associated with YAP in clinical breast cancer specimens (*R* = 0.382, *p* < 0.01). Gene correlation analysis was based on the TCGA breast invasive carcinoma dataset and was analysed via the R2: Genomics Analysis and Visualization Platform. The degrees of freedom (df) was 1095

To determine how YAP-TEAD regulates FAK phosphorylation, a collection of TEAD4 ChIP-sequence data (two replications, SL14575 and SL16341) in MCF7 cells was first obtained from the ENCODE database (GSM1010860) and analysed via the ChIP-Seek tool (Fig. 5a). Peaks located in the promoter-transcription start site (TSS) region were exacted and annotated. As shown in Fig. 5b, a total of 192 genes whose promoter was potentially combined with TEAD4 were identified. Next, gene expression profiling was performed to confirm the mRNA expression levels of these 192 genes in MCF7 cells overexpressing the YAP-S127A mutant (Fig. 5c). Among them, 30 genes were upregulated in the YAP-S127A mutant (fold change greater than 2) (Fig. 5d). To further identify the potential upstream genes of FAK which could be transcriptionally activated by YAP-TEAD, gene ontology (GO) enrichment analysis of these 30 upregulated genes was performed. As shown in Fig. 5e, “cell adhesion” was represented as the first GO enrichment category and included 6 genes (THBS1, HABP2, L1CAM, BCAM, CYR61 and CTGF). Interestingly, the “cell adhesion” category also ranked 6th in the GO enrichment analysis of all upregulated genes (1416 genes) that were identified in the YAP-S127A mutant in MCF7 cells (Additional file 4 Table S3). Through the String program, THBS1 appeared to be the potential upstream regulatory factor of FAK (Fig. 5f).

Thrombospondin 1 (THBS1) has been widely reported to be an activator of FAK Tyr397 phosphorylation [26–28]. According to the ChIP-sequence data from the ENCODE database, TEAD4 can bind to the promoter region of THBS1 in MCF7 cells (Additional file 5: Fig. S2). To characterize the transcriptional regulation of THBS1 by YAP, a luciferase-based reporter containing the promoter region of THBS1 (TSS: -2000 ~ + 50 bp) was constructed and co-transfected with pcDNA3.1-YAP and Renilla plasmids into HEK293T and MCF7 cells. The dual luciferase reporter assay revealed that YAP overexpression could significantly increase THBS1 promoter activity (Fig. 5g). Subsequently, YAP-S127A and YAP-S94A mutants were co-transfected, and the results showed that compared with the YAP1-S94A mutant, YAP1-S127A could significantly promote THBS1 promoter activities in both HEK293T and MCF7 (Fig. 5h). Chromatin immunoprecipitation showed that overexpression of the YAP-S127A mutant led to an increased binding between YAP and the THBS1 promoter in MCF7 cells (Fig. 5i). Next, quantitative real-time PCR was performed to validate whether YAP could regulate THBS1 mRNA expression. Compared to YAP-S94A, overexpression of the YAP-S127A mutant significantly increased YAP target genes (CTGF and CYR61) [35] and THBS1 mRNA levels in MCF7 cells (Fig. 5j). Meanwhile, knockdown of endogenous YAP significantly inhibited both YAP target genes (CTGF and CYR61) and THBS1 expression in MDA-MB-231 cells (Fig. 5k). We further verified that the YAP-S127A mutant could upregulate THBS1 protein levels in MCF7 cells via Western blot assay (Fig. 5l). Furthermore, either knockdown of YAP expression or disrupting the YAP-TEAD complex with verteporfin could significantly inhibit THBS1 protein expression in MDA-MB-231 cells (Fig. 5m, n). Collectively, these data demonstrated that THBS1 was the target gene of YAP and could be transcriptionally activated by the YAP-TEAD complex. Finally, to validate the expression correlation between YAP and THBS1 in clinical breast cancer specimens, the TCGA database (breast invasive carcinoma dataset) was used and analysed via the R2: Genomics Analysis and Visualization Platform (http://r2.amc.nl). As shown in Fig. 5o, THBS1 expression was positively associated with YAP expression in breast cancer (*R* = 0.382, *p* < 0.001).

### YAP triggers FAK phosphorylation and focal adhesion through THBS1

**Fig. 6:**
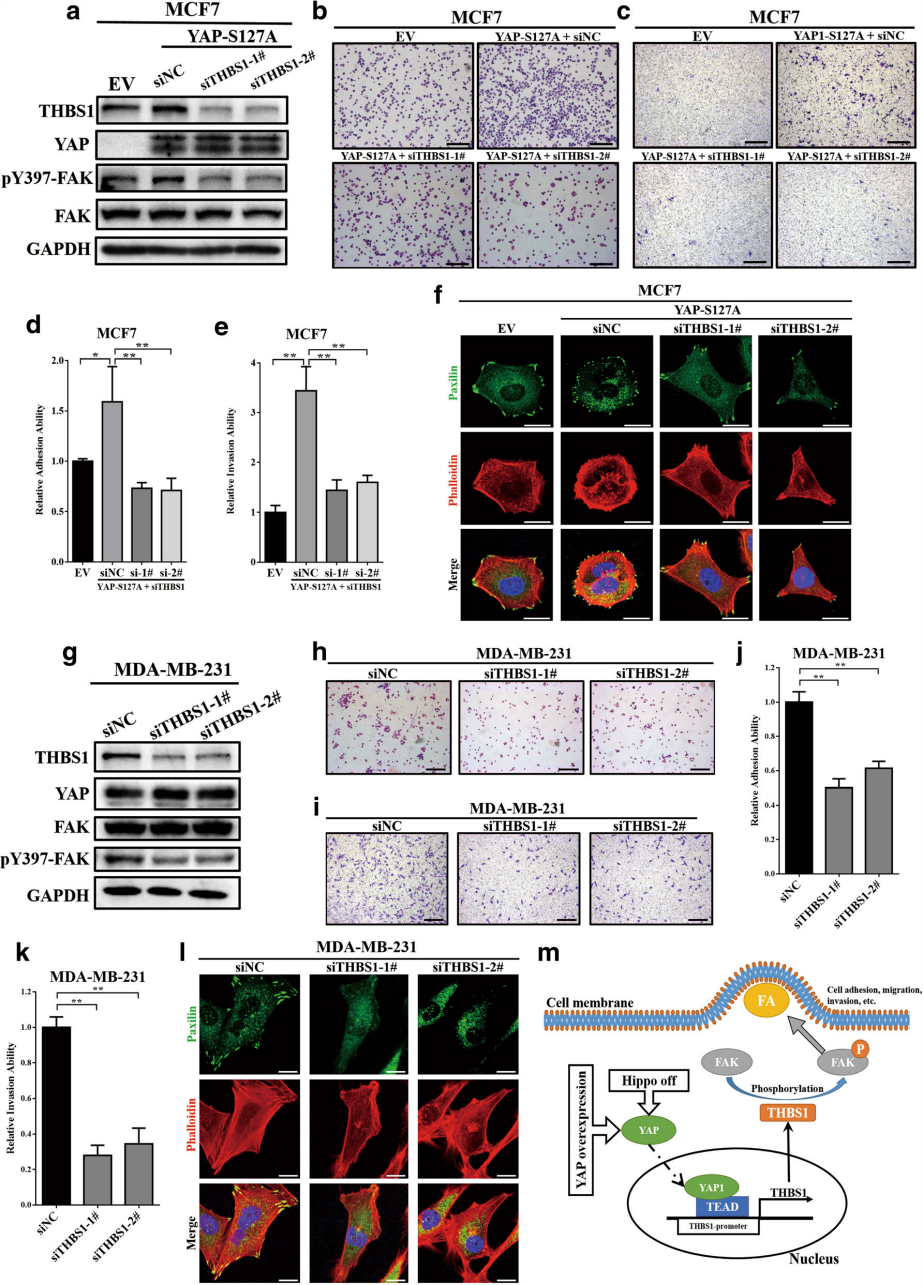
YAP triggered FAK phosphorylation and focal adhesion through THBS1. (**a**) Western blot assays revealed that knockdown of THBS1 expression in MCF7-YAP-S127A cells could significantly reverse FAK Y397 phosphorylation. (**b**) Cell adhesion assays showed that knockdown of THBS1 could significantly reverse YAP-S127A-induced cell adhesion in MCF7 cells. The experiments were performed in triplicate. Scale bar: 100 μm. (**c**) Transwell invasion assays showed that knockdown of THBS1 could significantly reverse YAP-S127A-induced cell invasion in MCF7 cells. The experiments were performed in triplicate. Scale bar: 100 μm. (**d**) Quantification of the cell adhesion ability in (**b**). * *p* < 0.05 and ** *p* < 0.01 by ANOVA test. (**e**) Quantification of the cell invasion ability in (**c**). ** *p* < 0.01 by ANOVA test. (**f**) Knockdown of THBS1 inhibited focal adhesion in MCF7-YAP-S127A cells. Red: F-actin (stained with phalloidin); Green: paxilin; Blue: nucleus (stained with DAPI). Scale bar: 20 μm. (**g**) Knockdown of THBS1 reduced FAK Y397 phosphorylation in MDA-MB-231 cells. (**h**) Knockdown of THBS1 expression reduced cell adhesion to gelatin in MDA-MB-231 cells. The experiments were performed in triplicate. Scale bar: 100 μm. (**i**) Transwell invasion assays showed that knockdown of THBS1 expression reduced cell invasion in MDA-MB-231 cells. The experiments were performed in triplicate. Scale bar: 100 μm. (**j**) Quantification of the cell adhesion ability in (**h**). ** *p* < 0.01 by ANOVA test. (**k**) Quantification of the cell invasion ability in (**i**). ** *p* < 0.01 by ANOVA test. (**l**) Knockdown of THBS1 reduced focal adhesion in MDA-MB-231 cells. Red: F-actin (stained with phalloidin); Green: paxilin; Blue: nucleus (stained with DAPI). Scale bar: 20 μm. (m) Model for how YAP regulates THBS1 expression and induces focal adhesion

Previous experiments have proven that YAP could transcriptionally promote THBS1 expression. To reveal the role of THBS1 in YAP-induced FAK phosphorylation and focal adhesion, a collection of siRNAs targeting THBS1 was used to knockdown THBS1 expression in MCF7-YAP-S127A cells. As shown in Fig. 6a, knockdown of THBS1 expression reversed FAK-Tyr397 phosphorylation in MCF7-YAP-S127A cells. In addition, cell adhesion assays and transwell invasion assays showed that knockdown of THBS1 expression could also inhibit the YAP-S127A-induced cell adhesion and invasion ability in MCF7 cells (Fig. 6b-6e). Through immunofluorescence, we observed that the number of focal adhesions was significantly reduced when MCF7-YAP-S127A cells were transfected with THBS1 siRNAs (Fig. 6f). In MDA-MB-231 cells, similarly with YAP expression knockdown, silencing THBS1 could reduce FAK phosphorylation (Fig. 6g), inhibit cell adhesion and invasion abilities (Fig. 6h-6k), and decrease focal adhesions (Fig. 6l). This demonstrates that YAP triggers FAK phosphorylation and focal adhesion through THBS1.

In summary, these results revealed a model in which YAP regulated FAK Tyr397 phosphorylation through transcriptionally activating THBS1 expression and induced focal adhesion and cell invasion in breast cancer (Fig. 6m).

## Discussion

Previous studies have reported that the dysregulation of YAP is highly associated with tumour aggressiveness and metastasis in breast cancer [22, 23, 40]; however a concrete mechanism for this remains unknown. In this research, we provide evidence from clinical specimens and breast cancer cell lines that YAP acts as a promoter of focal adhesion and tumour invasiveness via regulating the transcription of thrombospondin 1, leading to the phosphorylation of FAK (Fig. 6m). Our findings show that YAP could induce FAK activation in breast cancer cell lines in a TEAD-dependent manner, thus resulting in an increase of focal adhesion and tumour invasion. Using gene expression profiling and bioinformatics analysis, we identified the FAK upstream gene, thrombospondin 1, as a direct transcriptional target of YAP-TEAD. Further experiments haven proven that silencing of THBS1 could reverse YAP-induced FAK activation and focal adhesion. These findings reveal a new signal axis, YAP/THBS1/FAK, in the modulation of cell adhesion and invasiveness and provide new insights into how the Hippo pathway regulates tumour metastasis in breast cancer. Consequently, interfering in this signal axis could be an efficient way to inhibit breast cancer cell invasion and metastasis.

It is well known that YAP plays a critical role in cancer development and progression [20]. In breast cancer, YAP is often reported as an oncogene, and its hyper-activation often leads to various tumour-promoting effects [41, 42]. Previous studies have reported the association between YAP and breast cancer cell aggressiveness [22]. Furthermore, the overexpression of YAP has been shown to be a trigger of epithelial–mesenchymal transition [43] and actin dynamics [24]. As the main effector of the Hippo pathway, activation of YAP is controlled by Hippo signalling [19, 20]. Hippo signalling is an evolutionarily conserved regulator of tissue growth and cell fate and is mainly regulated by the actin cytoskeleton and cellular tension [44, 45]. Recent studies have revealed numerous upstream signalling mechanisms involved in the Hippo pathway, including cell polarity, mechanotransduction and G protein-coupled receptor signalling [45]. As a key component of cell-ECM crosstalk, focal adhesions have been demonstrated to play an important role in cellular mechanotransduction [6] and act as a link between integrin and Hippo signalling [45] in tumours. In the conventional viewpoint, FAK activation and focal adhesion appear to be an upstream signalling mechanism that triggers Hippo-off and induces YAP activation signalling [46]. However, a recent study has unveiled that YAP could directly control the RhoA GTPase pathway to induce FA assembly in AD-MSC and CAL51 cells [47]. Therefore, it is reasonable that YAP could promote tumour metastasis in an FA-dependent manner.

In our current study, we observed that YAP induces focal adhesions in breast cancer cells. Through gene expression screening and bioinformatics analysis, we discovered a new signalling axis, YAP/THBS1/FAK, in Hippo-mediated cell adhesion and invasion. THBS1 was the first member to be identified in the thrombospondins family and is a main player in the tumour microenvironment [48]. THBS1 was demonstrated decades ago to be a cell adhesion protein [49]. Thereafter, numerous studies have proven that THBS1 regulates cell adhesion in different cell types, regardless of species [48]. Previous studies have shown that THBS1 could modulate FAK phosphorylation to regulate focal adhesion dynamics [26, 27, 50, 51]. In addition, increased expression of THBS1 has also been reported to be associated with tumour invasiveness and metastasis [52–54]. Due to its essential role in tumour progression, THBS1 represents a perspective therapeutic target in cancer treatment. However, very little is known about its upstream regulation. Our current results reveal a novel mechanism where YAP induces FAK phosphorylation via activating THBS1 transcription in a TEAD-dependent manner. These findings reveal a new crosstalk mechanism between the Hippo pathway and THBS1-FAK signalling and provide a new interpretation of YAP-regulated tumour aggressiveness in breast cancer.

Numerous reports have shown that the FAK gene is amplified in a large fraction of breast cancer specimens; meanwhile, increased FAK expression and activity frequently correlates with metastatic disease and poor prognosis (reviewed in [15]). FAK has been demonstrated to play an important role in the progression of tumour aggressiveness; thus, it has been selected as a potential target for cancer therapeutics [18]. Several FAK inhibitors (GSK2256098, VS-4718, VS-6062, defactinib, and BI853520) have been entered into clinical trials, and some have achieved promising clinical activities in patients with selected solid cancers [18, 55]. Although inhibition of FAK has shown effectiveness in the control of cancer, little is known regarding the predictive response biomarkers of FAK-targeting agents. In our research, we have shown a correlation between Hippo signalling and FAK activation; therefore, YAP overexpression may act as a potential biomarker of FAK inhibitor treatment in breast cancer.

In summary, with this study, we have proven that YAP acts as an upstream regulator in focal adhesion dynamics and have discovered a YAP/THBS1/FAK signalling mechanism in the regulation of cell invasiveness and adhesion in breast cancer. These findings reveal a new role of Hippo signalling in focal adhesion in breast cancer and provide exciting opportunities for future studies.

## Conclusions

In this study, we offer evidences that YAP promotes focal adhesion and tumour invasiveness in breast cancer. Moreover, we unveil a new signal axis, YAP/THBS1/FAK, in the modulation of cell adhesion and invasiveness, and provides new insights into the crosstalk between Hippo signalling and focal adhesion (Fig. 6m). These findings reveal a new role of Hippo signalling in focal adhesion in breast cancer and provide exciting opportunities for future studies.

## Additional files

Additional file 1: **Table S1.** The sequences of siRNAs used in this research. (DOC 32 kb)

Additional file 2: **Table S2.** Primer sequences used in this research. (DOC 38 kb)

Additional file 3: **Figure S1.** Gene set enrichment analysis (GSEA) of purified tumour cells from 14 primary breast tumour tissues and 6 metastatic lymph nodes from the GEO database (GSE30480). C6: oncogenic gene sets were used in this analysis. ES: enrichment score; NES: normalized enrichment score; NOM-p: normalized *p*-value; FDR-q: false discovery rate q-value; FWER-p: family-wise error rate *p*-value. (JPG 17399 kb)

Additional file 4: **Table S3.** Gene ontology enrichment (biological processes) of all upregulated genes (1416 genes with fold change greater than 1.5) affected by the YAP-S127A mutant in MCF7 cells. “Cell adhesion” was the 6th enrichment category and contained 30 genes. Gene categories were ranked by –Log_10_P value. The categories with *p* > 0.01 were omitted from this table. (DOC 82 kb)

Additional file 5: **Figure S2.** The binding sequence of TEAD4 to the THBS1 gene. SL14575 and SL16341 were two bio-replications of the TEAD4 ChIP-sequence data from the ENCODE database. Sequence data were mapped to NCBI GRCh37 (hg19) according to the protocol and analysed via the ChIP-seek tool. The TEAD4 binding site was calculated as the aggregate of the TEAD4 binding peaks from the two bio-replicates. TSS: transcription start site. (JPG 986 kb)

## Abbreviations

ATCC: American Type Culture Collection
ChIP: Chromatin immunoprecipitation
CTGF: Connective tissue growth factor
CYR61: Cysteine-rich angiogenic inducer 61
DAPI: 4′,6-diamidino-2-phenylindole
DAVID: The Database for Annotation, Visualization and Integrated Discovery
DMEM: Dulbecco’s modified Eagle medium
DMSO: Dimethyl sulfoxide
ECM: Extracellular matrix
ENCODE: Encyclopaedia of DNA Elements
FA: Focal adhesion
FAK: Focal adhesion kinase (also known as PTK2, protein tyrosine kinase 2)
GAPDH: Glyceraldehyde-3-phosphate dehydrogenase
GFP: Green fluorescent protein
GSEA: Gene set enrichment analysis
IHC: Immunohistochemistry
L15: Leibovitz’s L-15 medium
qRT-PCR: Quantitative real-time PCR
SDS-PAGE: Sodium dodecyl sulfate polyacrylamide gel electrophoresis
TCGA: The Cancer Genome Atlas
TEAD: TEA domain family member
THBS1: Thrombospondin 1
TSS: Transcription start site
YAP: Yes-associated protein
