# Correction: Hippo component YAP promotes focal adhesion and tumour aggressiveness via transcriptionally activating THBS1/FAK signaling in breast cancer

**DOI:** 10.1186/s13046-023-02864-1

**Published:** 2023-10-20

**Authors:** Jie Shen, Beibei Cao, Yatao Wang, Chenshen Ma, Zhuo Zeng, Liang Liu, Xiaolan Li, Deding Tao, Jianping Gong, Daxing Xie

**Affiliations:** 1 Molecular Medicine Center, Tongji Hospital, Tongji Medical College, Huazhong University of Science and Technology, 430030 Wuhan, People’s Republic Of China; 2 Department of Surgery, Tongji Hospital, Tongji Medical College, Huazhong University of Science and Technology, 430030 Wuhan, People’s Republic Of China

**Correction: *J Exp Clin Cancer Res*** **37, 175 (2018)**

10.1186/s13046-018-0850-z

Following the publication of the original article [1], errors were found in Figs. 2i and 6i. Incorrect adhesion assay photo of “siYAP-2#” group into “siYAP-3#” group in Fig. 2i and the Transwell photo of “siTHBS1-1#” group into the “siTHBS1-2#” group in Fig. 6i were presented.

The authors declare that the correction does not change the results or conclusions of this paper. The correct figures are given below:

**Fig. 2:**
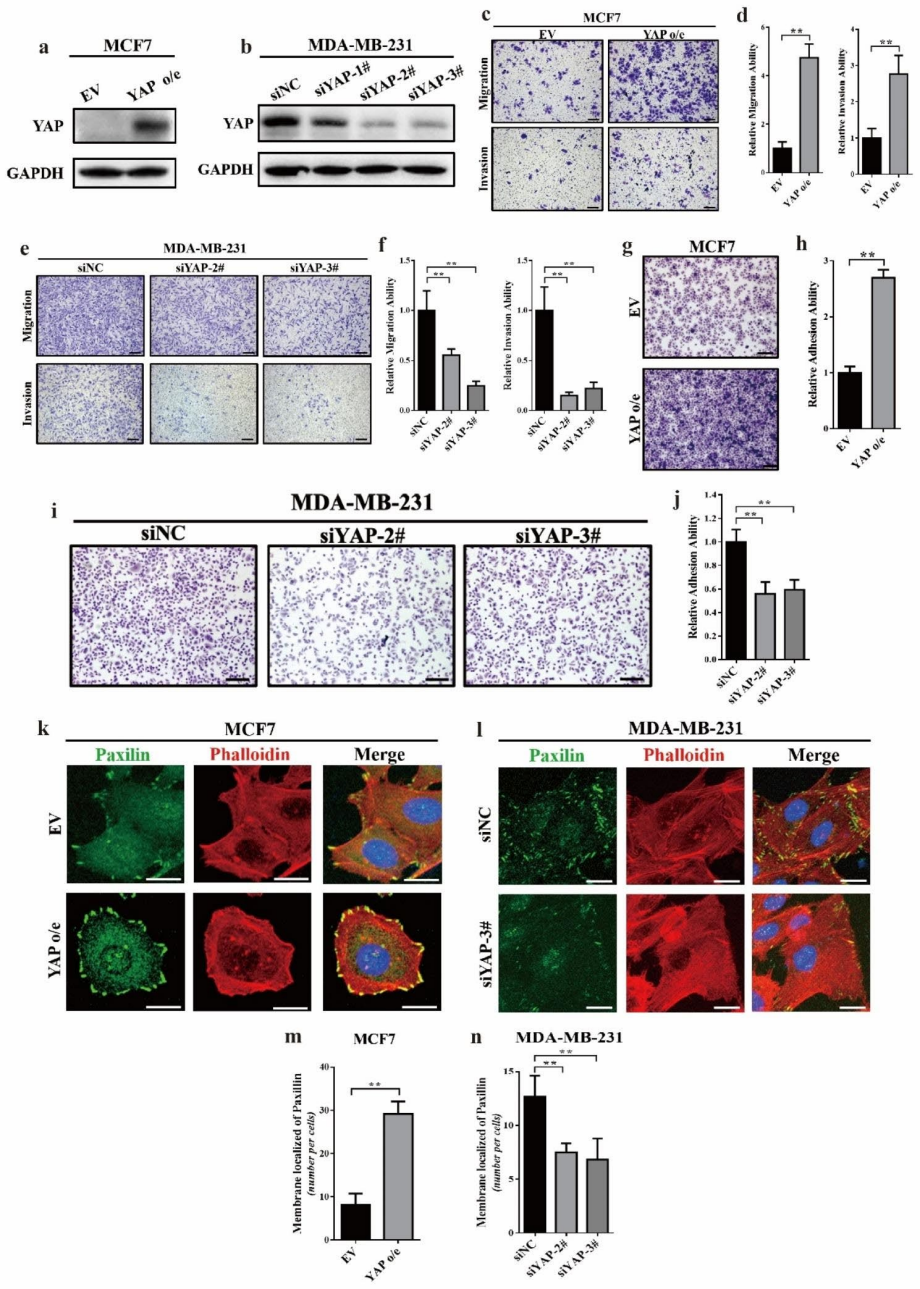
YAP was able to induced cell migration, invasion and focal adhesion in breast cancer cell lines. (**a**) Western blot verified the overexpression of YAP in MCF7 cells. EV: empty vector; o/e: overexpression. (**b**) Western blot verified the knockdown of YAP in MDA-MB-231 cells via a collection of siRNAs; siYAP-#2 and siYAP-#3 has relatively high knockdown efficiency, thus these two siRNAs were used in this research. (**c, d**) Transwell assay showing that overexpression of YAP induced cell migration and invasion ability in MCF7 cells. The experiment was performed in triplicate. **p < 0.01 by Student’s t-test. Scale bar: 100 μm. (**e, f**) Transwell assay showing that knockdown of YAP significantly inhibited cell migration and invasion ability in MDA-MB-231 cells. The experiment was performed in triplicate. ** *p* < 0.01 by ANOVA test. Scale bar: 100 μm. (**g, h**) Overexpression of YAP induced MCF7 cell adhesion to gelatin. The attached cells were stained with Wright’s-Giemsa and are shown in (**g**). The experiment was performed in triplicate. ** *p* < 0.01 by Student’s t-test. Scale bar: 100 μm. (**i, j**) Knockdown of YAP significantly inhibited MDA-MB-231 cell adhesion to gelatin. The attached cells were stained with Wright’s-Giemsa and are shown in (**i**). The experiment was performed in triplicate. ** *p* < 0.01 by Student’s t-test. Scale bar: 100 μm. (**k**) Overexpression of YAP induced focal adhesions in MCF7 cells. Focal adhesions were visualized by co-localization of paxilin (green) and F-actin (stained with phalloidin, red). Nuclei were counterstained with DAPI (blue). Scale bar: 20 μm. (**l**) Knockdown of YAP expression inhibited focal adhesions in MDA-MB-231 cells. Scale bar: 20 μm. (**m**) Quantification of the membrane-localized paxilin in (**k**). The experiment was performed in triplicate. ** *p* < 0.01 by Student’s t-test. (**n**) Quantification of the membrane-localized paxilin in (**l**). The experiment was performed in triplicate. ** *p* < 0.01 by ANOVA test

**Fig. 6:**
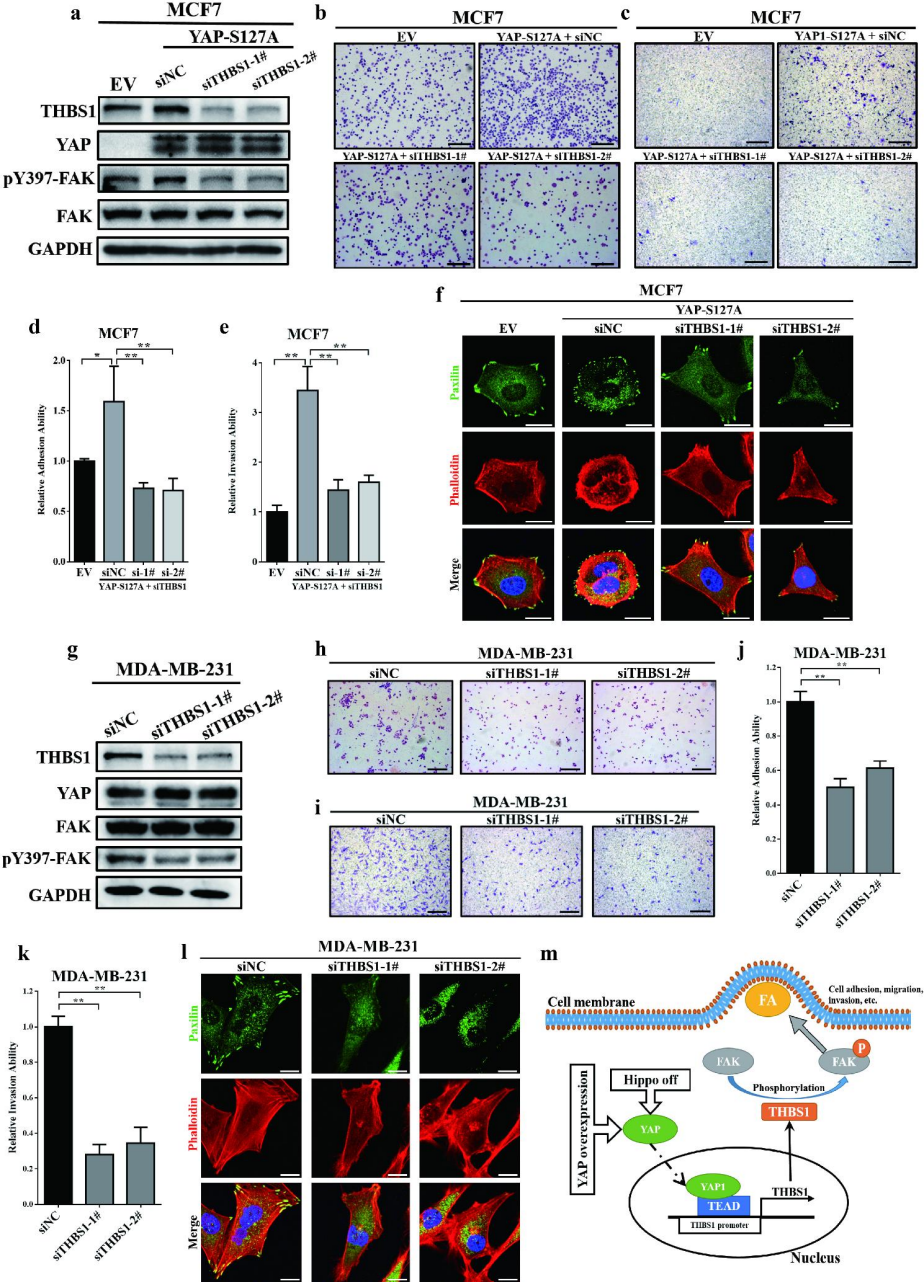
YAP triggered FAK phosphorylation and focal adhesion through THBS1. (**a**) Western blot assays revealed that knockdown of THBS1 expression in MCF7-YAP-S127A cells could significantly reverse FAK Y397 phosphorylation. (**b**) Cell adhesion assays showed that knockdown of THBS1 could significantly reverse YAP-S127A-induced cell adhesion in MCF7 cells. The experiments were performed in triplicate. Scale bar: 100 μm. (**c**) Transwell invasion assays showed that knockdown of THBS1 could significantly reverse YAP-S127A-induced cell invasion in MCF7 cells. The experiments were performed in triplicate. Scale bar: 100 μm. (**d**) Quantification of the cell adhesion ability in (**b**). * *p* < 0.05 and ** *p* < 0.01 by ANOVA test. (**e**) Quantification of the cell invasion ability in (**c**). ** *p* < 0.01 by ANOVA test. (f) Knockdown of THBS1 inhibited focal adhesion in MCF7-YAP-S127A cells. Red: F-actin (stained with phalloidin); Green: paxilin; Blue: nucleus (stained with DAPI). Scale bar: 20 μm. (**g**) Knockdown of THBS1 reduced FAK Y397 phosphorylation in MDA-MB-231 cells. (**h**) Knockdown of THBS1 expression reduced cell adhesion to gelatin in MDAMB-231 cells. The experiments were performed in triplicate. Scale bar: 100 μm. (**i**) Transwell invasion assays showed that knockdown of THBS1 expression reduced cell invasion in MDA-MB-231 cells. The experiments were performed in triplicate. Scale bar: 100 μm. (**j**) Quantification of the cell adhesion ability in (**h**). ** *p* < 0.01 by ANOVA test. (**k**) Quantification of the cell invasion ability in (**i**). ** *p* < 0.01 by ANOVA test. (**l**) Knockdown of THBS1 reduced focal adhesion in MDA-MB-231 cells. Red: F-actin (stained with phalloidin); Green: paxilin; Blue: nucleus (stained with DAPI). Scale bar: 20 μm. (**m**) Model for how YAP regulates THBS1 expression and induces focal adhesion
