# Supplementary material for: Hippo component YAP promotes focal adhesion and tumour aggressiveness via transcriptionally activating THBS1/FAK signalling in breast cancer
Source: J Exp Clin Cancer Res. 2018 Jul 28;37:175. doi: 10.1186/s13046-018-0850-z (PMC6064138; doi:10.1186/s13046-018-0850-z)
Supplement: Supplementary file 1 — Table S1. The sequences of siRNAs used in this research. (DOC 32 kb) [file 13046_2018_850_MOESM1_ESM.doc]

**Target sequences of siRNAs**

| **Name** | **Target Sequence (5`-3`)** |
| --- | --- |
| si-YAP1-1# | CAGTGGCACCTATCACTCT |
| si-YAP1-2# | GCGTAGCCAGTTACCAACA |
| si-YAP1-3# | GGTGATACTATCAACCAAA |
| si-THBS1-1# | GCGTGTTTGACATCTTTGA |
| si-THBS1-2# | CTGCGTTGGTGATGTAACA |
|  |  |
|  |  |
|  |  |
|  |  |
|  |  |
