# Supplementary material for: Hippo component YAP promotes focal adhesion and tumour aggressiveness via transcriptionally activating THBS1/FAK signalling in breast cancer
Source: J Exp Clin Cancer Res. 2018 Jul 28;37:175. doi: 10.1186/s13046-018-0850-z (PMC6064138; doi:10.1186/s13046-018-0850-z)
Supplement: Supplementary file 2 — Table S2. Primer sequences used in this research. (DOC 38 kb) [file 13046_2018_850_MOESM2_ESM.doc]

**Primers` sequences**

| **Primer** | **Sequence (5`-3`)** |
| --- | --- |
| GAPDH-F | CTCCTGCACCACCAACTGCT |
| GAPDH-R | GGGCCATCCACAGTCTTCTG |
| YAP-F | TAGCCCTGCGTAGCCAGTTA |
| YAP-R | TCATGCTTAGTCCACTGTCTGT |
| CTGF-F | AGGAGTGGGTGTGTGACGA |
| CTGF-R | CCAGGCAGTTGGCTCTAATC |
| CYR61-F | CCTTGTGGACAGCCAGTGTA |
| CYR61-R | ACTTGGGCCGGTATTTCTTC |
| THBS1-F | AGACTCCGCATCGCAAAGG |
| THBS1-R | TCACCACGTTGTTGTCAAGGG |
|  |  |
|  |  |
|  |  |
|  |  |
|  |  |
|  |  |
|  |  |
|  |  |
|  |  |
|  |  |
|  |  |
|  |  |
