# Supplementary figures and images for: Hippo component YAP promotes focal adhesion and tumour aggressiveness via transcriptionally activating THBS1/FAK signalling in breast cancer
Source: J Exp Clin Cancer Res. 2018 Jul 28;37:175. doi: 10.1186/s13046-018-0850-z (PMC6064138; doi:10.1186/s13046-018-0850-z)

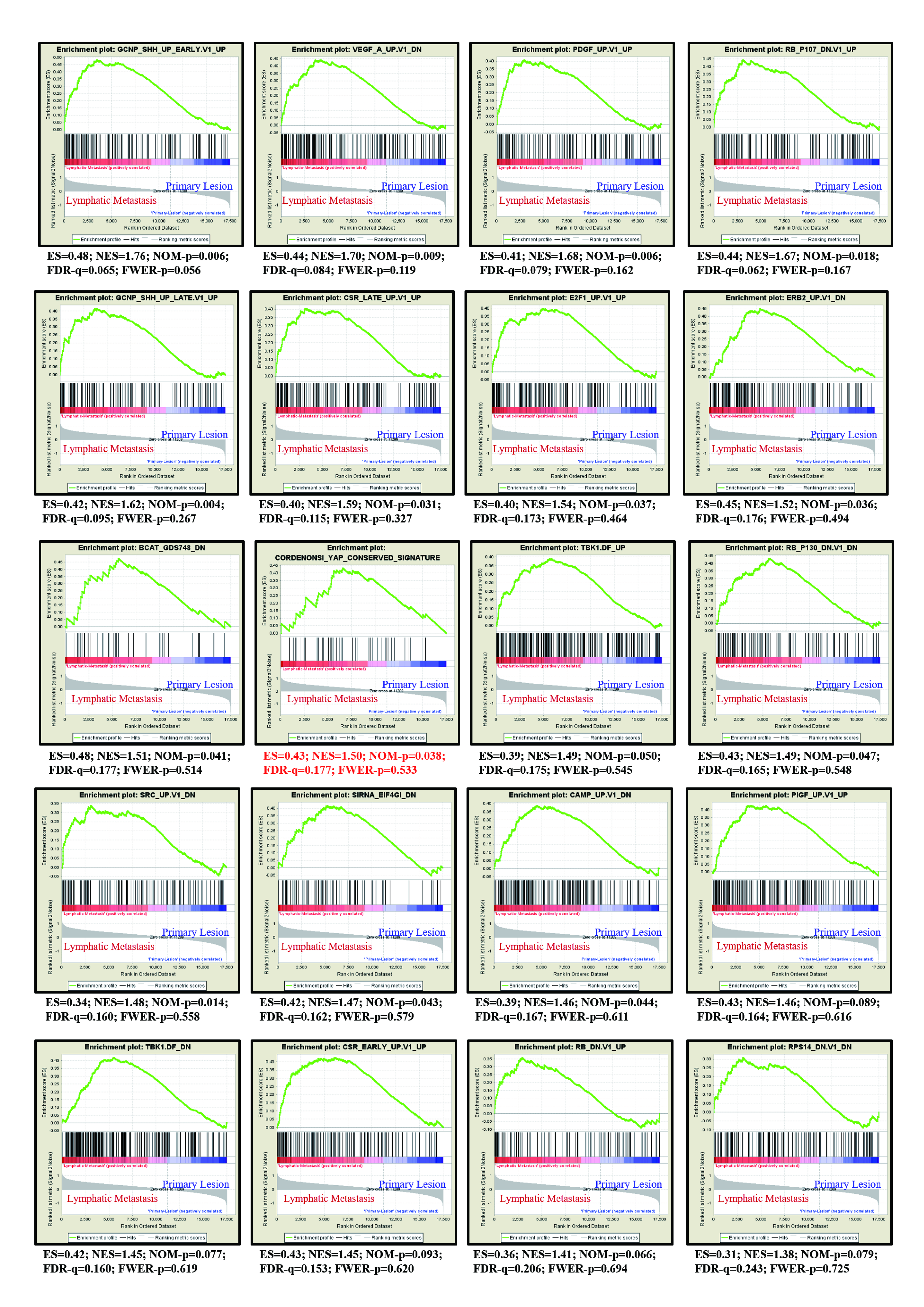

Supplement: Supplementary file 3 — Figure S1. Gene set enrichment analysis (GSEA) of purified tumour cells from 14 primary breast tumour tissues and 6 metastatic lymph nodes from the GEO database (GSE30480). C6: oncogenic gene sets were used in this analysis. ES: enrichment score; NES: normalized enrichment score; NOM-p: normalized p-value; FDR-q: false discovery rate q-value; FWER-p: family-wise error rate p-value. (JPG 17399 kb) [file 13046_2018_850_MOESM3_ESM.jpg]

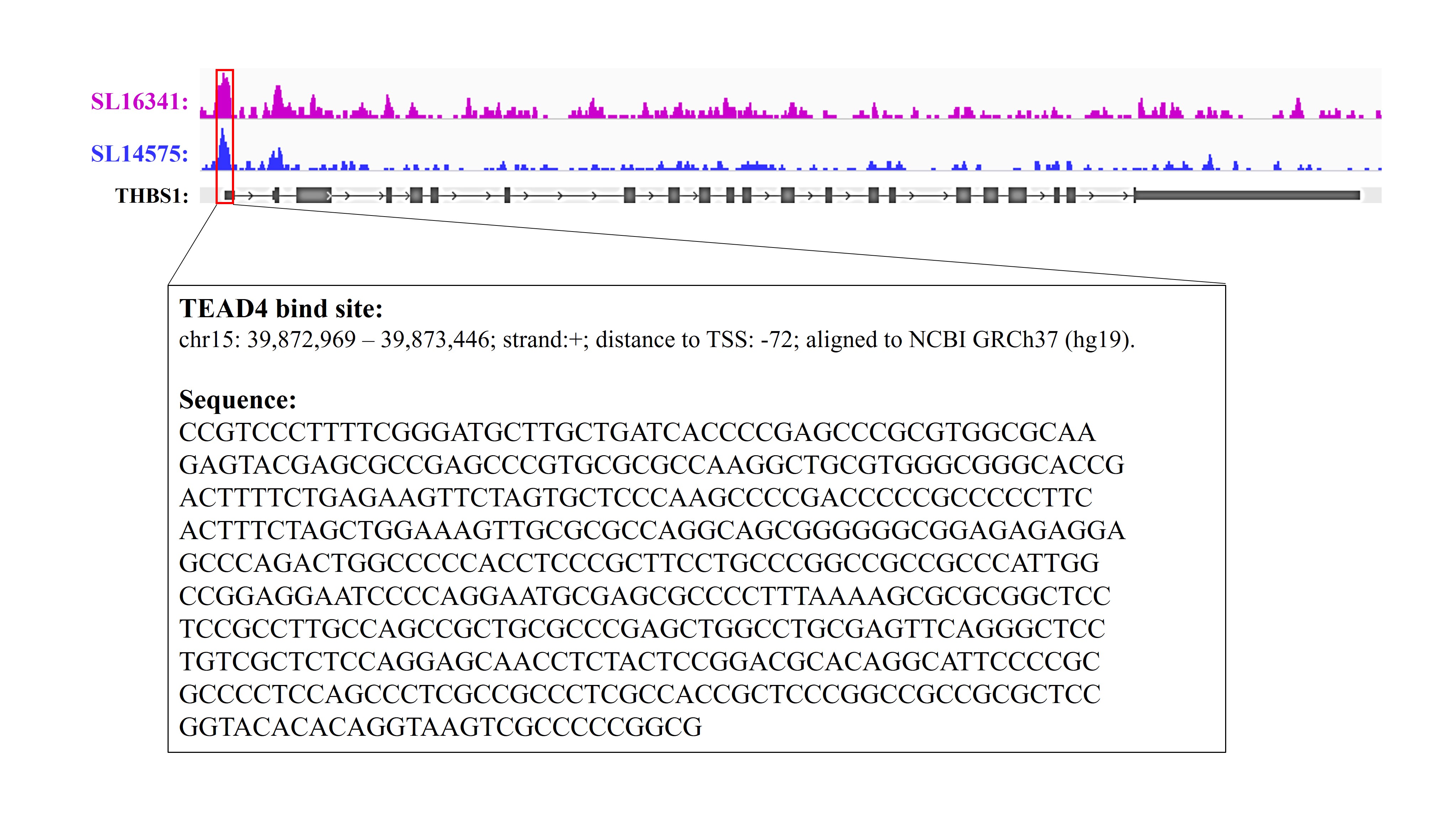

Supplement: Supplementary file 5 — Figure S2. The binding sequence of TEAD4 to the THBS1 gene. SL14575 and SL16341 were two bio-replications of the TEAD4 ChIP-sequence data from the ENCODE database. Sequence data were mapped to NCBI GRCh37 (hg19) according to the protocol and analysed via the ChIP-seek tool. The TEAD4 binding site was calculated as the aggregate of the TEAD4 binding peaks from the two bio-replicates. TSS: transcription start site. (JPG 986 kb) [file 13046_2018_850_MOESM5_ESM.jpg]
