# Supplementary material for: Hippo component YAP promotes focal adhesion and tumour aggressiveness via transcriptionally activating THBS1/FAK signalling in breast cancer
Source: J Exp Clin Cancer Res. 2018 Jul 28;37:175. doi: 10.1186/s13046-018-0850-z (PMC6064138; doi:10.1186/s13046-018-0850-z)
Supplement: Supplementary file 4 — Table S3. Gene ontology enrichment (biological processes) of all upregulated genes (1416 genes with fold change greater than 1.5) affected by the YAP-S127A mutant in MCF7 cells. “Cell adhesion” was the 6th enrichment category and contained 30 genes. Gene categories were ranked by –Log10P value. The categories with p > 0.01 were omitted from this table. (DOC 82 kb) [file 13046_2018_850_MOESM4_ESM.doc]

| **Rank** | **Term_description** | **Gene Count** | **P_value** | **-log10(pvalue)** |
| --- | --- | --- | --- | --- |
| 1 | DNA methylation on cytosine | 11 | 5.3284E-08 | 7.27340302 |
| 2 | chromatin silencing at rDNA | 11 | 2.6315E-07 | 6.579794631 |
| 3 | DNA replication-dependent nucleosome assembly | 10 | 2.8372E-07 | 6.547109791 |
| 4 | protein heterotetramerization | 10 | 5.6821E-07 | 6.245492896 |
| 5 | histone H4-K20 demethylation | 6 | 3.1655E-05 | 4.499563272 |
| 6 | cell adhesion | 30 | 3.2795E-05 | 4.48419246 |
| 7 | negative regulation of megakaryocyte differentiation | 6 | 5.4311E-05 | 4.265112725 |
| 8 | negative regulation of gene expression, epigenetic | 11 | 0.00024511 | 3.610643827 |
| 9 | DNA replication-independent nucleosome assembly | 6 | 0.0002994 | 3.523755448 |
| 10 | nucleosome assembly | 13 | 0.00031585 | 3.500517112 |
| 11 | homophilic cell adhesion via plasma membrane adhesion molecules | 14 | 0.00038365 | 3.416061734 |
| 12 | regulation of gene expression, epigenetic | 11 | 0.00062994 | 3.200697487 |
| 13 | regulation of gene silencing | 4 | 0.00078419 | 3.105577091 |
| 14 | small GTPase mediated signal transduction | 28 | 0.00094828 | 3.02306447 |
| 15 | metanephric epithelium development | 3 | 0.0012741 | 2.894797464 |
| 16 | smoothened signaling pathway | 8 | 0.0013447 | 2.871376183 |
| 17 | sex determination | 3 | 0.00186947 | 2.728280428 |
| 18 | regulation of metanephric nephron tubule epithelial cell differentiation | 3 | 0.00186947 | 2.728280428 |
| 19 | calcium-dependent cell-cell adhesion via plasma membrane cell adhesion molecules | 5 | 0.00226807 | 2.644344066 |
| 20 | metanephric collecting duct development | 3 | 0.00261253 | 2.58293817 |
| 21 | positive regulation of peroxisome proliferator activated receptor signaling pathway | 3 | 0.00261253 | 2.58293817 |
| 22 | CENP-A containing nucleosome assembly | 6 | 0.00272556 | 2.564543898 |
| 23 | calcium ion transmembrane transport | 11 | 0.00289742 | 2.537987809 |
| 24 | positive regulation of neuron projection development | 8 | 0.00305757 | 2.514623574 |
| 25 | cytoskeleton organization | 10 | 0.00307705 | 2.511865313 |
| 26 | glycosphingolipid biosynthetic process | 3 | 0.00351414 | 2.454180577 |
| 27 | positive regulation of smooth muscle contraction | 4 | 0.00422143 | 2.374540416 |
| 28 | regulation of synaptic plasticity | 5 | 0.00435224 | 2.361286995 |
| 29 | synapse assembly | 6 | 0.00450745 | 2.346069403 |
| 30 | protein O-linked glycosylation | 8 | 0.00502972 | 2.298455846 |
| 31 | negative regulation of activated T cell proliferation | 3 | 0.00582982 | 2.234344608 |
| 32 | type B pancreatic cell development | 3 | 0.00582982 | 2.234344608 |
| 33 | female pregnancy | 8 | 0.0065264 | 2.185326169 |
| 34 | axon guidance | 21 | 0.00690845 | 2.160619612 |
| 35 | telomere maintenance | 7 | 0.0070063 | 2.154511073 |
| 36 | mesonephros development | 3 | 0.00725922 | 2.139110123 |
| 37 | positive regulation of transforming growth factor beta receptor signaling pathway | 4 | 0.0086938 | 2.060790228 |
| 38 | positive regulation of organ growth | 3 | 0.00887798 | 2.051686079 |
| 39 | protein O-linked fucosylation | 3 | 0.00887798 | 2.051686079 |
| 40 | negative regulation of gene expression | 9 | 0.00956258 | 2.019424724 |
| 41 | pituitary gland development | 4 | 0.00985471 | 2.006355978 |
| 42 | negative regulation of Ras protein signal transduction | 4 | 0.00985471 | 2.006355978 |
